# Photoacoustic Imaging-Guided Photothermal Therapy with Tumor-Targeting HA-FeOOH@PPy Nanorods

**DOI:** 10.1038/s41598-018-27204-8

**Published:** 2018-06-11

**Authors:** Thi Tuong Vy Phan, Nhat Quang Bui, Soon-Woo Cho, Subramaniyan Bharathiraja, Panchanathan Manivasagan, Madhappan Santha Moorthy, Sudip Mondal, Chang-Seok Kim, Junghwan Oh

**Affiliations:** 1 Interdisciplinary Program of Biomedical Mechanical & Electrical Engineering, Pukyong National University, Busan, 48513 Republic of Korea; 2 Center for Marine-Integrated Biomedical Technology, Pukyong National University, Busan, 48513 Republic of Korea; 3 Department of Biomedical Engineering, Pukyong National University, Busan, 48513 Republic of Korea; 4 Department of Cogno-Mechatronics Engineering, Pusan National University, Busan, 46241 Republic of Korea

## Abstract

Cancer theragnosis agents with both cancer diagnosis and therapy abilities would be the next generation of cancer treatment. Recently, nanomaterials with strong absorption in near-infrared (NIR) region have been explored as promising cancer theragnosis agents for bio-imaging and photothermal therapy (PTT). Herein, we reported the synthesis and application of a novel multifunctional theranostic nanoagent based on hyaluronan (HA)-coated FeOOH@polypyrrole (FeOOH@PPy) nanorods (HA-FeOOH@PPy NRs) for photoacoustic imaging (PAI)-guided PTT. The nanoparticles were intentionally designed with rod-like shape and conjugated with tumor-targeting ligands to enhance the accumulation and achieve the entire tumor distribution of nanoparticles. The prepared HA-FeOOH@PPy NRs showed excellent biocompatible and physiological stabilities in different media. Importantly, HA-FeOOH@PPy NRs exhibited strong NIR absorbance, remarkable photothermal conversion capability, and conversion stability. Furthermore, HA-FeOOH@PPy NRs could act as strong contrast agents to enhance PAI, conducting accurate locating of cancerous tissue, as well as precise guidance for PTT. The *in vitro* and *in vivo* photothermal anticancer activity results of the designed nanoparticles evidenced their promising potential in cancer treatment. The tumor-bearing mice completely recovered after 17 days of PTT treatment without obvious side effects. Thus, our work highlights the great potential of using HA-FeOOH@PPy NRs as a theranostic nanoplatform for cancer imaging-guided therapy.

## Introduction

Breast cancer is the most frequent tumor among women in the world and the leading cause of cancer death in women^1,2^. Triple-negative breast cancer (TNBC) is a highly aggressive and malignant form of breast cancer and comes up with poor prognosis and limited treatment method options^3^. A subpopulation of breast cancer stem cells (CSCs) is resistant to conventional therapies and strongly contributes to the high ratio of recurrence and metastasis of TNBC^4^. CSCs are identified by the surface cell markers such as CD44 receptors with highly expression^5^. Targeting CSCs therapy has been considered as an efficient and powerful therapeutic strategy that can significantly reduce the relapse and metastasis^6^. Thus, this strategy has been highly focusing on the recent development of new therapies for the breast cancer treatment^7–9^.

Photothermal therapy (PTT) is emerging as a great therapeutic in the treatment of cancer metastasis which generates heat from near-infrared (NIR) light by photothermal agents^10^. Compared with the conventional cancer therapies, PTT exhibits unique advantages including high specificity to tumor, minimal invasiveness to surrounding normal tissues, and temporal and spatial selectivity^10,11^. Recently photoacoustic imaging (PAI) has emerged as a promising imaging technique for diagnostic and therapeutic monitoring purposes^12^. Exogenous imaging agents are used in PAI to further increase the contrast and specificity of imaging or target specific molecular processes^13^. Therefore, PAI with the assistance of tumor-targeting nanoplatforms can accurately locate the tumor for the more-precisely guided PTT.

Photothermal therapeutic agents should have strong absorption of NIR biological transparency window (700–1870 nm) to set the greater penetration depth into the solid tumor while reducing the scattered and absorbed photons by surrounding healthy tissues to minimize unwanted damages^14–17^. Over the past decade, many different types of NIR-absorbing agents have been developed for PTT such as metal-based nanomaterials [gold nanorods^18,19^, gold nanocages^20^, gold nanoshells^21^, copper sulfide nanocrystals^22^, iron oxide magnetic nanoparticles^23^, palladium nanosheets^24^], other inorganic nanomaterials [upconversion nanoparticles^25^], and organic nanomaterials [carbon nanotube^26^, graphene^27^, indocyanine green^28^, and polypyrrole nanoparticles (PPy NPs)^29,30^]. Among them, PPy NPs are considered powerful photothermal agents. They exhibit not only strong NIR absorbance, efficient photothermal conversion, biocompatibility, low cytotoxicity but also great photostability even under many repeated NIR irradiating times^29,31,32^. Additionally, PPy NPs can be easily synthesized with high yield and low cost^33^, which enable the convenience in fabrication process and decrement in treatment cost. Because of their strong NIR absorbance, PPy NPs were also used as PAI contrast agents for cancer diagnosis^34^. A number of research groups have employed PPy NPs *in vivo* imaging-guided cancer PTT^32,35^.

For targeting of tumors on cancer therapy, hyaluronan (HA) can be used as tumor-targeting molecules. TNBC cells have high expression of a CD44 protein which functions as a receptor for HA^36,37^. The structural studies showed that HA interacts with CD44 through hydrogen bonds and van der Waals forces^38^. Obviously, HA can be used as a tumor-targeting ligand for targeting tumors in cancer therapy. HA-based nanomaterials can achieve tumor targetability based on HA–CD44 receptor interactions and their *in vitro* and *in vivo* anti-tumor efficacies have been demonstrated^39–41^.

In the current work, we developed novel nanoparticles of hyaluronan-polypyrrole nanorods (HA-FeOOH@PPy NRs) for PAI-guided PTT to target CD44 positive breast cancer cells. The nanoparticles were fabricated in rod-like shape with the coating of PPy polymer and HA layer. In comparison with sphere shape of most reported nanoparticles, the rod-like shape nanoparticles can penetrate into solid tumor more rapidly and achieve higher accumulation^42,43^. We examined physicochemical characteristics, heating effect as well as the photothermal stability of the developed nanoparticles. Subsequently, cancer cells killing effects have been investigated in MBA-MB-231 breast cancer cells using MTT assay and double-staining method. Afterwards, we performed *in vivo* PTT experiments on a mice model with the guidance of PAI to confirm the inhibition of breast cancer growth. The experimental results showed that the as-prepared multifunctional nanoparticles exhibited precise tumor-targeted imaging for guiding PTT with the positive outcome on the mouse model, thus holding great promising for future theranostic applications.

## Results

### Synthesis and characterization of HA-FeOOH@PPy NRs

**Figure 1:**
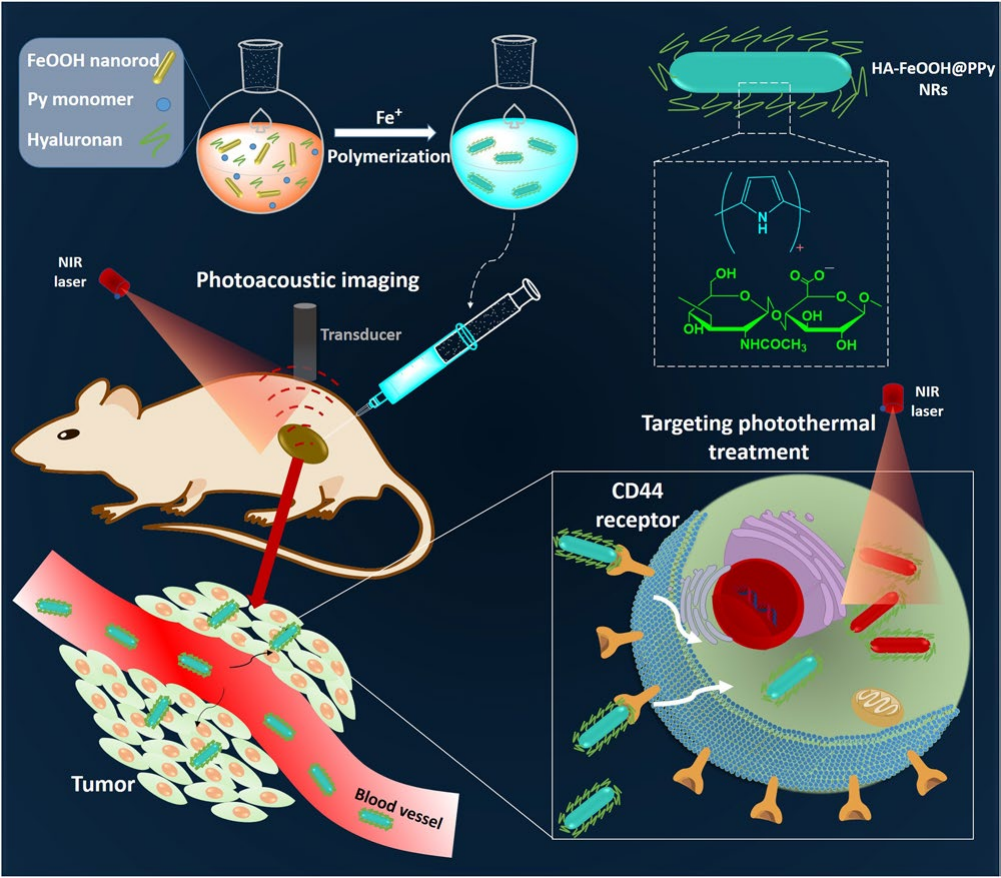
Schematic representation for the synthesis and the applications of HA-FeOOH@PPy NRs.

As illustrated in Fig. 1, the photothermal agents are delivered to the targeted tumor via the ligand-receptor interaction mechanism between HA on the nanoparticles and the CD44 receptor highly overexpressed on cancer cells; and the effective ablation of tumor is achieved by PTT with the guidance of PAI.

The synthesis procedure of HA-FeOOH@PPy NRs consisted of two steps. The first step was the preparation of monodisperse FeOOH nanorods which were used as rod-like templates for the synthesis of HA-FeOOH@PPy NRs, and the second step was the coating of PPy and HA to FeOOH nanorods.

**Figure 2:**
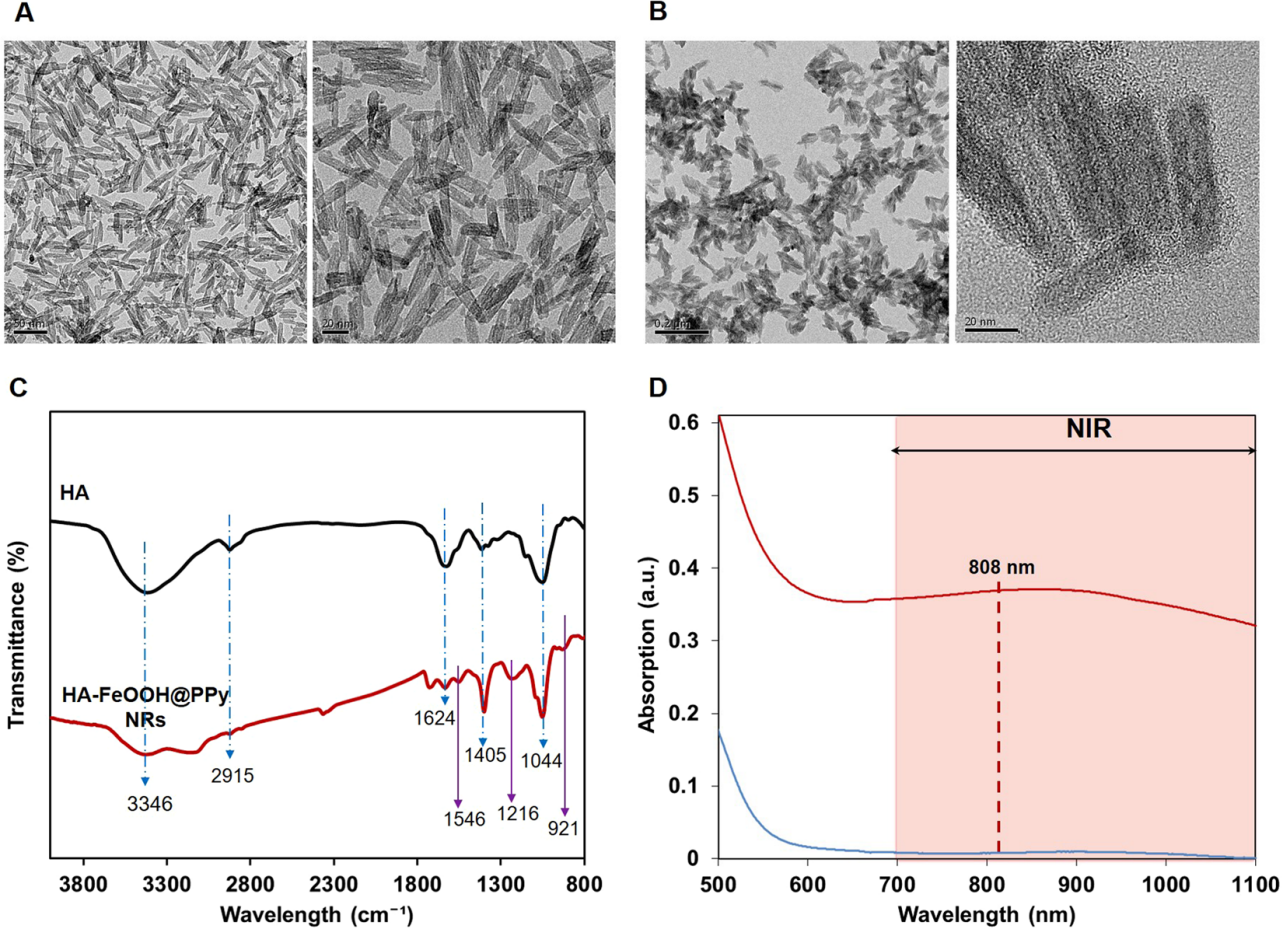
Characterization of nanoparticles. (**A**) FETEM images of FeOOH nanorods. (**B**) FETEM images of HA-FeOOH@PPy NRs. (**C**) FTIR of HA and HA-FeOOH@PPy NRs. (**D**) UV-Vis-NIR spectra of FeOOH nanorods and HA-FeOOH@PPy NRs.

As observed in Fig. 2A, the TEM images of FeOOH nanoparticles showed a uniform size of about 45 nm with rod-like shape. The coating of PPy polymer to the rod-like template was conducted via chemical oxidation polymerization using Fe^3+^ as an initiator agent. The formation of polypyrrole coating layer was indicated by the change of solution’s color from orange to black. The negative zeta potential (−11.63 mV) of HA-FeOOH@PPy NRs indicated the presence of negatively charged hyaluronan molecules on the surface of the nanoparticles (Table S1).

Figure 2C showed the FTIR spectra of HA and HA-FeOOH@PPy NRs. In the HA spectrum, the strong band at 3346 cm^−1^ was associated with the stretching vibration of OH group and the peak at 2915 cm^−1^ was related to CH_2_ group. The bands from 1624 cm^−1^ and 1405 cm^−1^ were respectively attributed the COOH group’s the symmetric (C–O) and asymmetric bending (C=O), while the peak at 1044 cm^−1^ was corresponding to the C–O–C hemiacetal system saccharide units. The identical peaks of HA which were clearly observed in the HA-FeOOH@PPy NRs spectrum indicated the successful coating of HA on the nanoparticles. The N–H stretching vibrations of PPy ring were related to the peak at 1546 cm^−1^. The peak at 1219 cm^−1^ was induced by the C-N stretching vibration and the peak at 921 cm^−1^ indicated the presence of polymerized pyrrole.

The UV-Vis spectra of FeOOH nanorods and HA-FeOOH@PPy NRs were observed using UV-Vis spectroscopy, as given in Fig. 2D. FeOOH nanorods had no absorption in NIR region, whereas the synthesized HA-FeOOH@PPy NRs showed a broadband absorption spectrum from UV, visible to NIR region. This indicated a successful coating of PPy layer. With the broadband absorption spectrum, HA-FeOOH@PPy NRs can be used flexibly to different demands.

The thermal stability of FeOOH rod-like templates, HA and HA-FeOOH@PPy NRs was analyzed by thermal gravity assay (TGA) (Fig. S1). It was observed that FeOOH rod-like templates were stable in the temperature range of 50 °C–800 °C, and just only 7% mass was lost at 800 °C. Pure HA showed that only 4% weight loss at temperature lower than 230 °C; however, the rapid decomposition occurred in the temperature range of 240 °C–800 °C, and only 32% mass remained at 800 °C. Similar to pure HA, some previous reports showed that dramatic decomposition was observed from 190 °C to 320 °C and just 30% mass remained at 800 °C for pure HA^44,45^. In contrast, more mass (about 65%) remained for HA-FeOOH@PPy NRs than pure PPy and HA at 800 °C. Because of FeOOH rod-like templates, HA-FeOOH@PPy NRs were thermally more stable than PPy and HA polymers. The high content of iron element made it easy to detect HA-FeOOH@PPy NRs in cell and tissues.

### Photothermal effects of HA-FeOOH@PPy NRs

**Figure 3:**
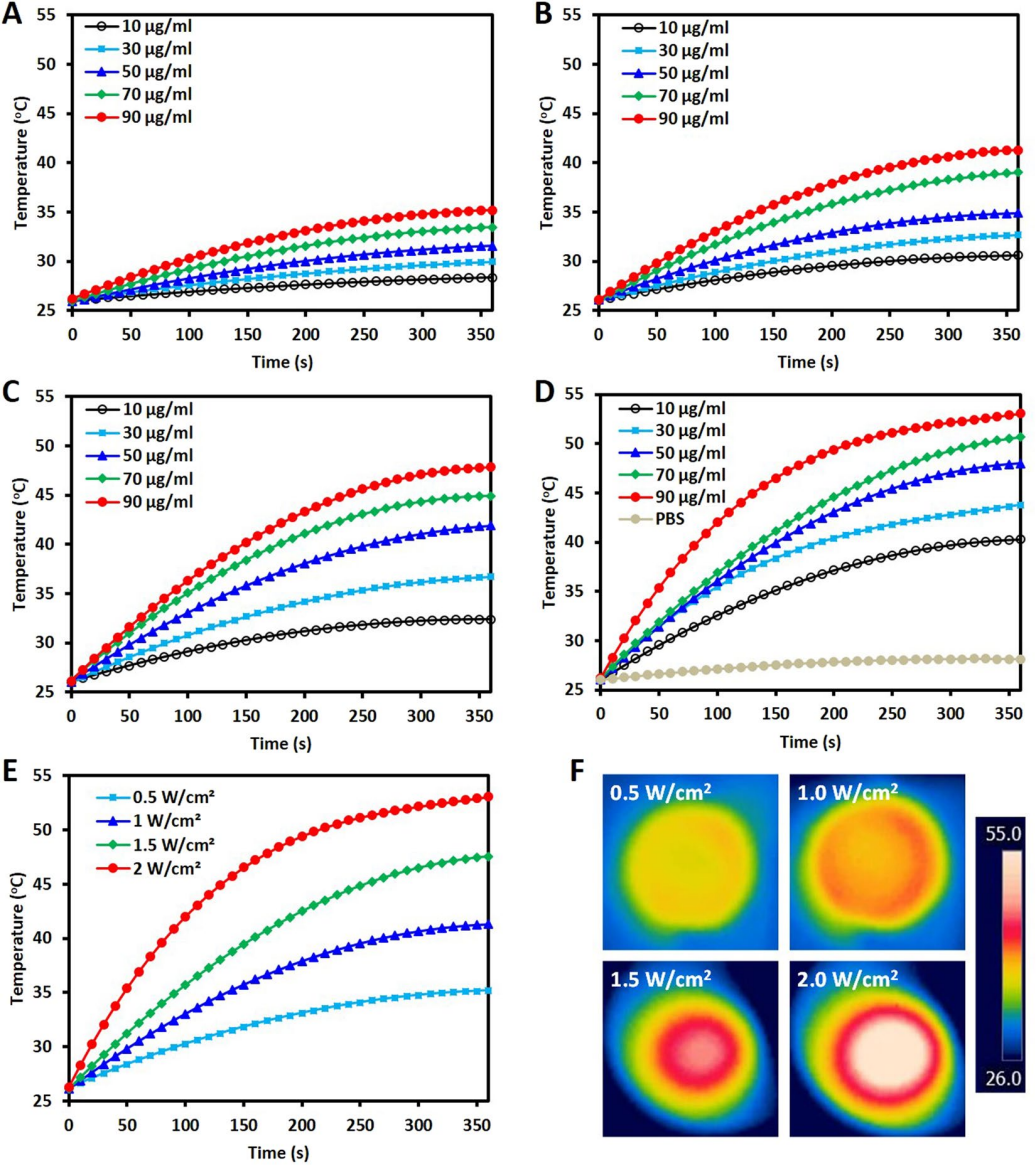
The heating effect evaluation of HA-FeOOH@PPy NRs. The solutions with different concentrations of HA-FeOOH@PPy NRs were irradiated with NIR laser for 6 min at different power densities of 0.5 W/cm^2^ (**A**), 1.0 W/cm^2^ (**B**), 1.5 W/cm^2^ (**C**), and 2.0 W/cm^2^ (**D**). (**E**) The temperature elevation of HA-FeOOH@PPy NRs solution (90 μg/mL) under NIR irradiation at different power densities (0.5 W/cm^2^, 1.0 W/cm^2^, 1.5 W/cm^2^, and 2.0 W/cm^2^) for 6 min. (**F**) The corresponding NIR thermographic images of the wells containing HA-FeOOH@PPy NRs (90 μg/mL) at the 360^th^ second.

The photothermal effect of HA-FeOOH@PPy NRs was evaluated by measuring the temperature elevation of HA-FeOOH@PPy NRs solution with different concentrations under NIR laser irradiation (0.5, 1.0, 1.5, and 2.0 W/cm^2^, 6 min), as presented in Fig. 3A–D. After 6 min of the NIR irradiation (2.0 W/cm^2^), the temperature increased to 53.1 °C for the HA-FeOOH@PPy NRs solution with the highest concentration of 90 µg/mL, whereas the temperature only increased to 41.1 °C for the HA-FeOOH@PPy NRs solution at the lowest concentration of 10 µg/mL. In contrast, the temperature of distilled water (DW) negligibly increased. As shown in Fig. 3E, at different laser power densities (0.5, 1.0, 1.5 and 2.0 W/cm^2^), the HA-FeOOH@PPy NRs solution (90 μg/mL) resulted in the temperature elevations of 35.2, 41.0, 47.7, and 53.1 °C, respectively. The thermographic images of all solutions at 360^th^ second are shown in Fig. 3F. The heating results indicated that the HA-FeOOH@PPy NRs could rapidly and efficiently convert the absorbed photon into heat and could serve as the photothermal agent for tumor ablation. These results also demonstrated that the NIR-heating behaviors of HA-FeOOH@PPy NRs were concentration-, power- and time-dependent.

The photothermal stability of nanoparticles is an important property for PTT. As shown in Fig. S3A, after three heating and cooling cycles under NIR laser irradiation (2 W/cm^2^), the thermal curve of HA-FeOOH@PPy NRs remained unchanged for each cycle; and the UV−Vis spectrum of HA-FeOOH@PPy NRs with no noticeable changes (Fig. S3B) suggested good photothermal stability after a long duration of laser irradiation.

The photothermal conversion efficiency (η) of the HA-FeOOH@PPy NRs was determined to be 37.17%, which is similar with that of the widely used CuS nanoparticles (38%, 980 nm)^46^ and relatively high compared with those of previously reported materials, such as the Au rods (∼21.0%, 810 nm laser^47^; 21.3%, 980 nm laser^46^), and Cu_9_S_5_ nanocrystals (∼25.7%, 980 nm laser)^48^.

The stability of photothermal agents under physiological conditions is a critical issue relating to their biomedical application. No significant changes were observed on the UV-Vis spectra of the HA-FeOOH@PPy NRs solution (Fig. S4A) after two months of storage and no aggregation was observed, which suggested the good stability in long-term storage. HA-FeOOH@PPy NRs also had good colloidal stability in different physiological solutions (Fig. S4B).

### Cell uptake and *in vitro* cell cytotoxicity assay

Free HA was used as a competitor with HA-FeOOH@PPy NRs on the intracellular cell uptake experiment. Prussian blue staining, which is used to demonstrate ferric iron and ferritin, was performed to detect the presence of HA-FeOOH@PPy NRs on cells because of the high content of iron element on the nanoparticles. The bright field images (Fig. S2) showed that HA-FeOOH@PPy NRs were quickly taken up by MDA-MB-231 cells within 2 h of incubation while free HA rapidly reduced the cellular uptake of HA-FeOOH@PPy NRs into the cells. This result demonstrated that HA-FeOOH@PPy NRs has good selectivity toward CD44 expressing MDA-MB-231 cell line due to the rod-like shape and the HA coating.

**Figure 4:**
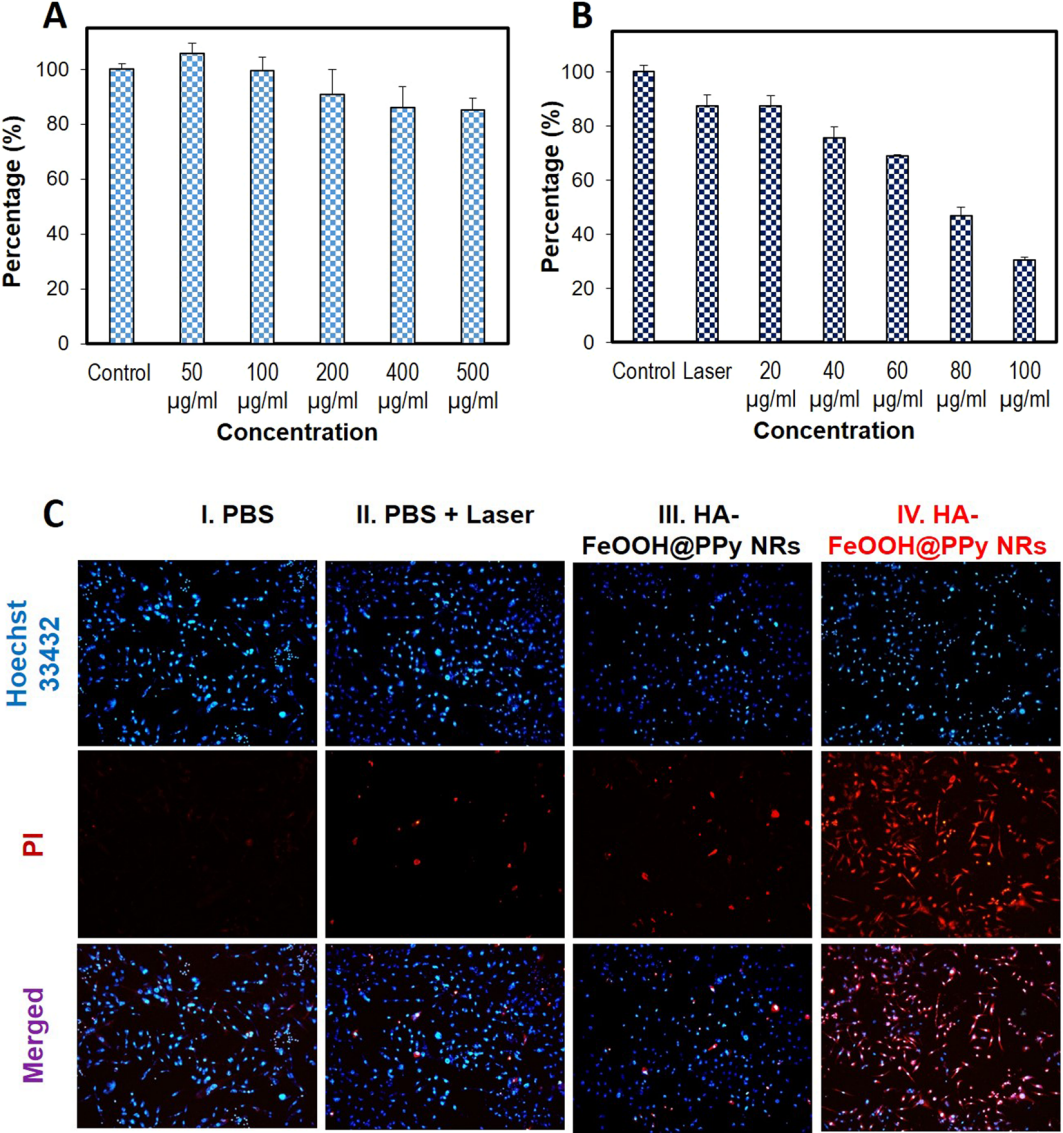
(**A**) The cell viability of MDA-MB-231 cells treated with HA-FeOOH@PPy NRs. (**B**) *In vitro* photothermal effects of HA-FeOOH@PPy NRs on MDA-MB-231 cells irradiated with NIR laser (2 W/cm^2^, 6 min). (**C**) Hochest and PI staining of MDA-MB-231 cells treated with PBS, laser only (2 W/cm^2^, 6 min), 100 µg/mL HA-FeOOH@PPy NRs, and 100 µg/mL HA-FeOOH@PPy NRs + laser (2 W/cm^2^, 6 min). Data are shown as the mean ± standard deviation (SD).

The biocompatible of HA-FeOOH@PPy NRs at the cellular level was determined using MTT assay. After 24 h incubation of MDA-MB-231 cells with HA-FeOOH@PPy NRs, no significant cytotoxicity was noticed even at 500 µg/mL, demonstrating good biocompatibility of HA-FeOOH@PPy NRs (Fig. 4A).

### Photothermal ablation of cancer cells *in vitro*

To evaluate the potential of HA-FeOOH@PPy NRs as photothermal agents, MTT assay was used to quantify the photothermal effect of HA-FeOOH@PPy NRs against MDA-MD-231 breast cancer cells. Quantitative analysis was conducted after NIR irradiation (2 W/cm^2^, 6 min) of MDA-MB-231 cells treated with various concentrations of HA-FeOOH@PPy NRs (20, 40, 60, 80, and 100 μg/mL) (Fig. 4B). The result of viability analysis showed that HA-FeOOH@PPy NRs plus NIR irradiation killed cells in a concentration-dependent behavior.

To further investigate the *in vitro* PTT effect of HA-FeOOH@PPy NRs, MDA-MD-231 cells were treated with HA-FeOOH@PPy NRs and then exposed to NIR laser at 2 W/cm^2^ for 6 min. Four groups were examined including phosphate buffered saline (PBS) (group I); PBS plus NIR laser (group II); HA-FeOOH@PPy NRs (group III); and HA-FeOOH@PPy NRs plus NIR laser (group IV). After treatment, the cells were co-stained with Hoechst 33342 and propidium iodide (PI) to evaluate the cell viability. Hoechst 33342 enters both live and dead cells and bind to DNA, emitting blue fluorescence; whereas PI only penetrates the damaged membrane of dead cells, emitting red fluorescence. The merged fluorescence image showed that the majority of cells on the groups I and II, III emitted green fluorescence, indicating negligible cell ablation. However, a large number of cells on group IV showed the red fluorescence, indicating almost cells were dead (Fig. 4C). The bright field images of MDA-MD-231 cells with trypan blue staining also showed the same results (Fig. S5). The boundary between blue and red fluorescence was clearly observed on the cell dish which was partly irradiated with laser spot (Fig. S6), further confirming that HA-FeOOH@PPy NRs could effectively kill the breast cancer cells exposed to NIR irradiation.

### *In vitro* and *in vivo* photoacoustic imaging

Tissue-mimicking PVA phantom was implanted with three wells of HA-PPy NR-treated MDA-MB-231 cells (50, 100, and 200 μg/mL) and one well of no treated cells as a control. The three wells containing HA-PPy NR-treated cells exhibited PA signals, whereas the control well did not show any PA signals (Fig. S7). High-amplitude PA signals were detected from the well with the highest HA-FeOOH@PPy NRs concentration. The *in vitro* PAI results suggested that HA-FeOOH@PPy NRs can be a good candidate for the photoacoustic agents. We afterward conducted *in vivo* PAI to demonstrate the ability of HA-FeOOH@PPy NRs as the nanoplatform for PAI-guided PTT.

**Figure 5:**
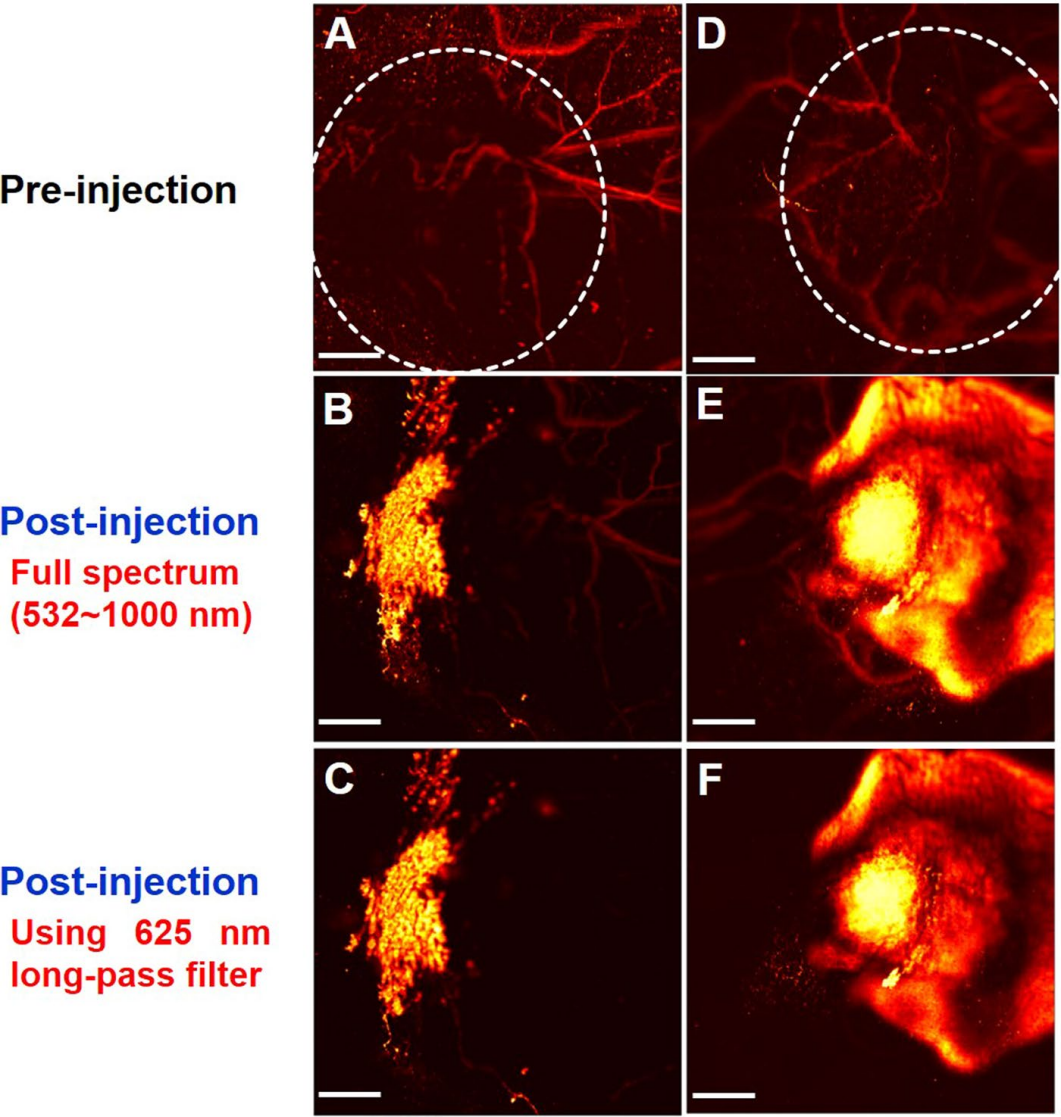
*In vivo* photoacoustic images of tumors. (**A**,**B**,**C**) The PAI of tumor before and after injection with FeOOH@PPy NRs and (**D**,**E**,**F**) the PAI of tumor before and after injection with HA-FeOOH@PPy NRs. Scale bar = 2 mm and applies to all images. White cycles indicate the tumor area.

Firstly, we considered PAI images of the tumor injected with HA-FeOOH@PPy NRs and FeOOH@PPy NRs (Fig. 5B–E). The PAI images showed good nanoparticle distribution with the injection of HA-FeOOH@PPy NRs, meanwhile, the FeOOH@PPy NRs remained localized only near the tumor center after 5 h of injection. The 625 nm long-pass filter revealed clearly the distribution of nanoparticles in the tumor. The entire tumor distribution of HA-FeOOH@PPy NRs can create a more uniform thermal profile and reach all points of the tumor. Thus, PTT using tumor-targeting HA-FeOOH@PPy NRs could be more effective than using non-targeting nanoparticles.

Secondly, we considered PAI images of the tumor before and after intratumoural injection of HA-FeOOH@PPy NRs. Before injection, only main blood vessels could be observed (Fig. 5C). After injection with HA-FeOOH@PPy NRs, the PAI imaging of tumor exhibited a clear PA signal with a significant increment in amplitude (Fig. 5D) in comparison with the same location before the injection, indicating the strong effect of HA-FeOOH@PPy NRs in generating PA signals. The distribution of HA-FeOOH@PPy NRs in the tumor can afford the enhanced and clear PAI imaging of tumor of live mice model. Hence, the detailed structure of tumor region could be acquired, ensuring the precise position of the external NIR laser. These observations confirmed that HA-FeOOH@PPy NRs can serve as photoacoustic contrast agents for the cancer theranostics using photoacoustic microscopy (PAM) system.

### *In vivo* photothermal therapy

**Figure 6:**
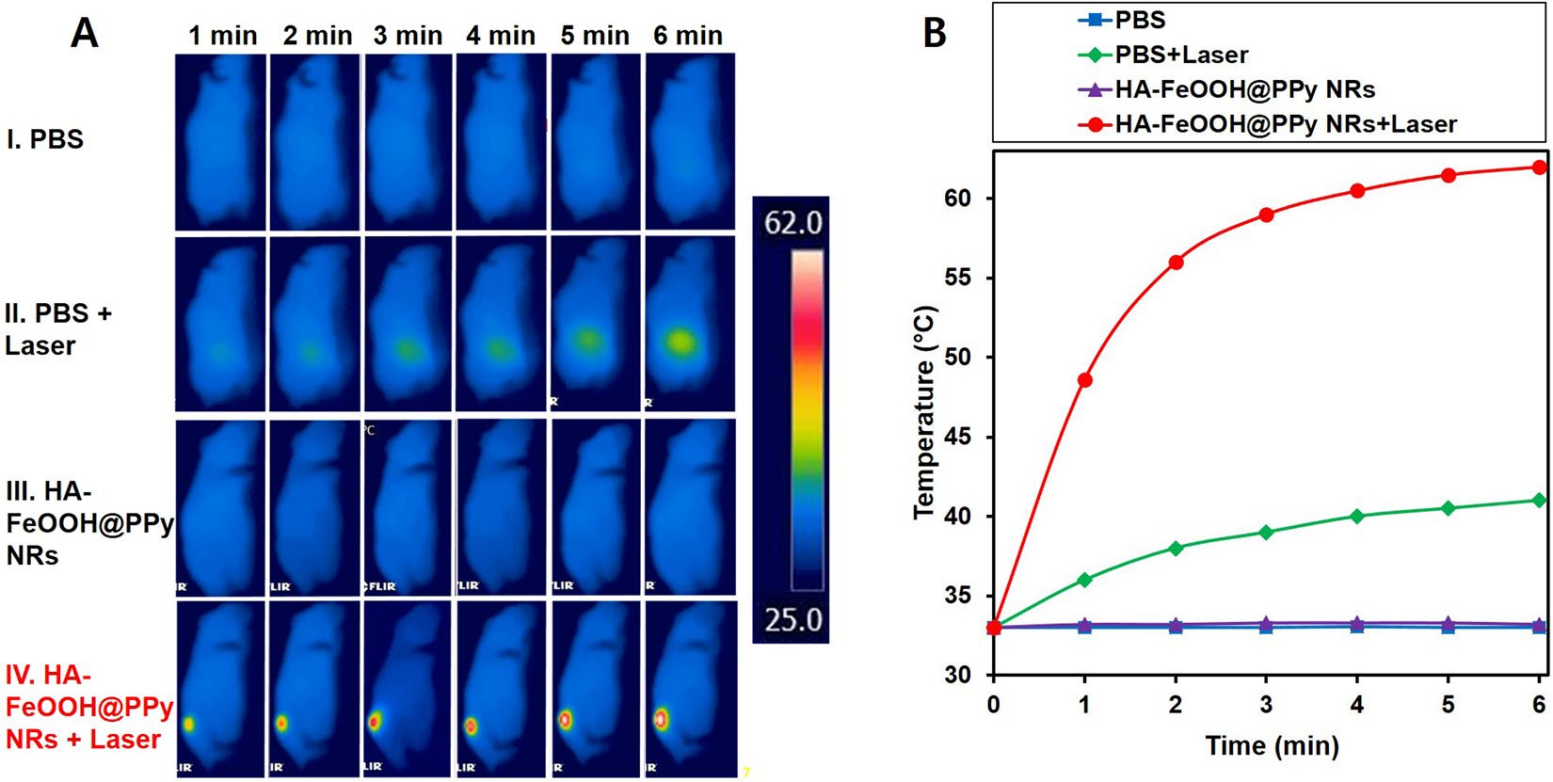
(**A**) Infrared thermograph map. (**B**) Temperature increasing in the tumor-bearing mice during 6 min irradiation by NIR laser at 2 W/cm^2^ after 5 h of PBS and HA-FeOOH@PPy NRs injection.

**Figure 7:**
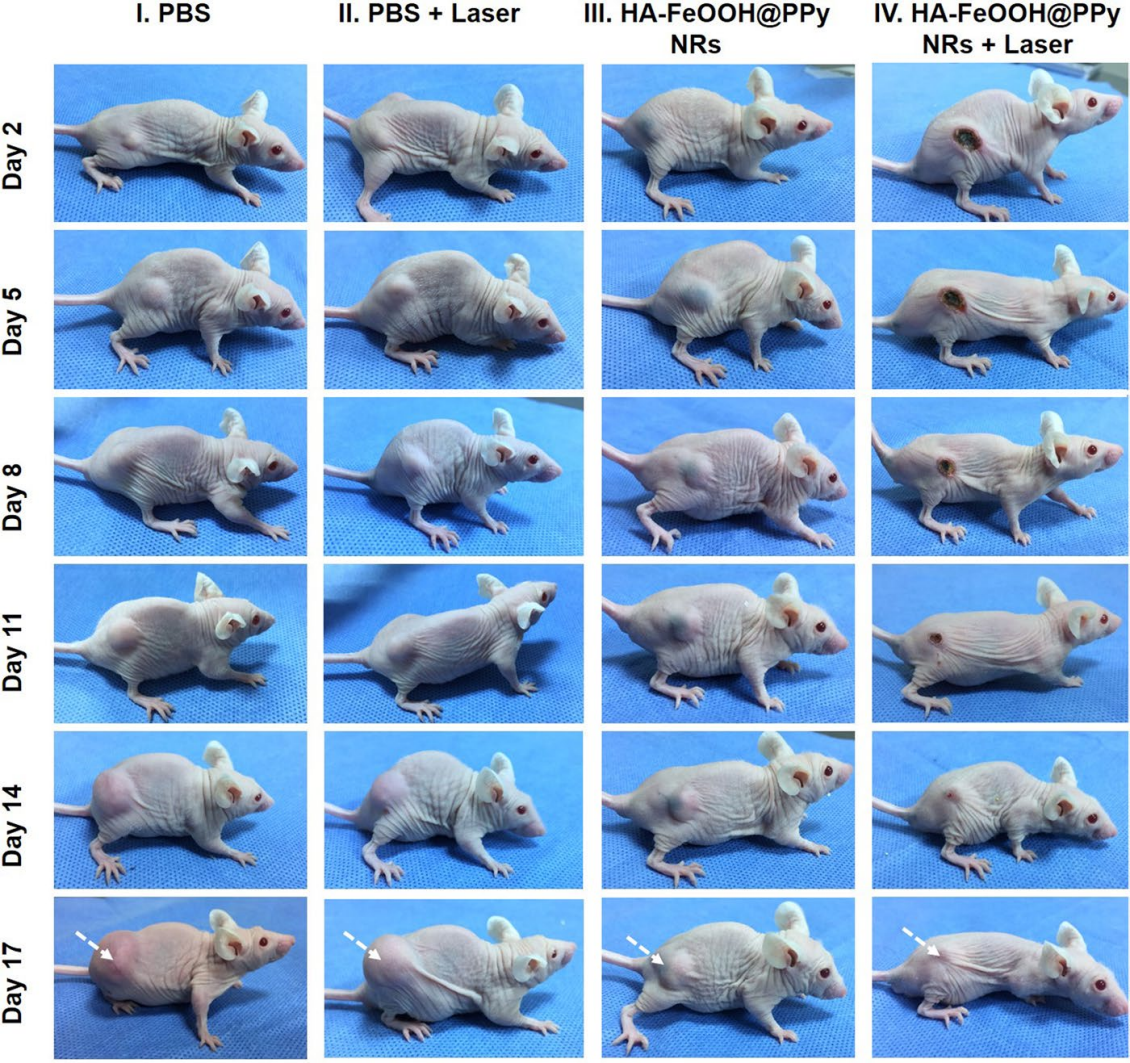
Representative photographs of tumor-bearing mice at day 2, 5, 8, 11, 15, and 17 after treatments of HA-FeOOH@PPy NRs plus 808 nm NIR laser irradiation (2.0 W/cm^2^, 6 min).

**Figure 8:**
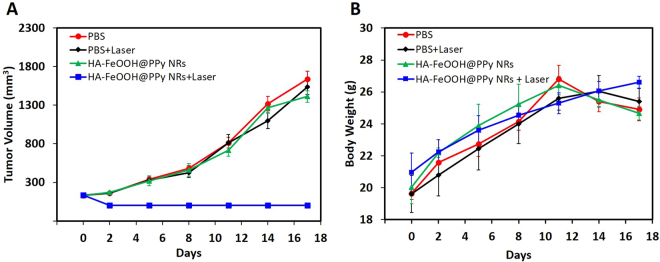
(**A**) Tumor volume growth of tumor-bearing mice of different groups during treatment period. (**B**) Body weight of tumor-bearing mice of different groups during treatment period. Data are shown as the mean ± SD.

To evaluate the ability of HA-FeOOH@PPy NRs in cancer treatment, *in vivo* PTT was further conducted on tumor mouse model. The tumor-bearing mice were randomly divided into four groups (n = 3) including PBS (group I), PBS + laser (group II), HA-FeOOH@PPy NRs (group III), and HA-FeOOH@PPy NRs + laser (group IV). 5 h after injection, the tumor-bearing mice of groups II and IV were anesthetized and the tumors were exposed the NIR laser at a power of 2 W/cm^2^ for 6 min (Fig. S8). To monitor the *in vivo* photothermal conversion effect of HA-FeOOH@PPy NRs, temperature changes on the tumor’s surface were recorded by a thermal camera. As shown in Fig. 6, the temperature of the tumor areas of mice from group IV could rapidly increase to about 62 °C within 6 min, which would cause an irreversible damage to the tissues. In contrast, the maximum average temperature of the tumor’s surface of mice on the group III was less than 34 °C, indicating that NIR laser itself could not sufficiently damage tumors. To verify the photothermal effect of HA-FeOOH@PPy NRs, tumor volume growth and body weight of the mice were monitored during the observation period. The tumors after the single treatment with the NIR laser irradiation only (group II) as well as HA-FeOOH@PPy NRs injection only (group III) continued to proliferate on the following days, as shown in Fig. 7 and Fig. 8A. The relative tumor volume after 17 days growth was about 11 times bigger than the initial tumor volume for the groups I, II, and III. However, HA-FeOOH@PPy NRs-mediated PTT could successfully inhibit the tumor growth and complete eradication of tumors after 17 days of treatment (Fig. S8). No obvious body weight loss in group IV was found (Fig. 8B), indicating that HA-FeOOH@PPy NRs-mediated PTT did not cause any significant toxicity in the treated mice. The tumors of mice from group IV were completely removed and no metastatic tumors were observed. Histological analysis (Fig. S10) also revealed no noticeable toxic effect of HA-FeOOH@PPy NRs on the major organs of mice after 17 days of photothermal treatment. These findings highlight that HA-FeOOH@PPy NRs can act as efficient PTT agents to convert the NIR light to sufficient thermal therapy for the ablation of tumor *in vivo*.

## Discussion

As is known to all, PTT utilizes the light-to-heat ability of photothermal agents that gain a lot of advantages in comparison with surgical resection, radiotherapy, and chemotherapy. However, on the way of translating the photothermal agents into the clinic, PTT faces a big challenge of obtaining heating of the entire tumor mass while avoiding unwanted damages to surrounding healthy tissue^49^. To achieve a homogeneous photothermal agent’s distribution, the photothermal agents need to be designed to easily penetrate into the entire tumor without expanding in surrounding healthy tissues. Thus, the nanomaterials with effective photothermal conversion properties as well as excellent *in vivo* tumor targeting need to be highly focused on the development of cancer therapy. In this work, we intentionally designed the rod-shape nanoparticles with tumor-targeting conjugated ligands to enhance the penetration of nanoparticles and achieve the entire tumor distribution of nanoparticles.

In general, nanoparticles in the bloodstream can be achieved targeting tumors by passive targeting (i.e., through the enhanced permeability and retention (EPR) effect) and/or active targeting (i.e., based on ligand-targeted tumor)^50,51^. However, intravenously injected nanoparticles often tend to home in the EPR organs, in particular, the liver, spleen, and lungs and they may cause long-term harmful effects^52^. The hypoxic areas of tumors that lack blood flow cause difficulties on the access of nanoparticles^53^. To avoid the inefficiencies and off-target depositions which have been observed from intravenous administration, direct intratumoural injections can be used alternatively^54–56^. In this work, we therefore prefer to use the intratumoural injections than intravenous injections on *in vivo* experiment on a mouse model.

PPy NPs are effective photothermal agents in the diagnosis and treatment of cancer due to their high NIR laser absorption. PPy NPs have previously been reported as potential photoacoustic contrast agents for imaging of deep tissues^34^. The high yield of PPy NPs can be easily achieved in the laboratory with low price^33^. A number of studies have examined PPy NPs for *in vitro* and *in vivo* imaging-guided PTT^32,35^. Herein, we utilized the PPy polymer to design theragnosis agents for PAI-guided PTT on cancer treatment.

Recently, HA has been highly focused on the designing of tumor-targeting nanoparticles. The simulation studies about structural model showed that HA binds strongly to CD44 via hydrogen bonds and van der Waals interaction^38^. A number of research groups have shown that CD44-targeted nanoparticles could be effectively bound onto CD44 overexpressed tumor cells such as HA/PEG-Gelatin/EGCG NPs for treatment of prostate tumor^39^, HA-coated chitosan NPs for anticancer drug delivery^57^, HA on monodisperse magnetite nanocrystals for targeted cancer imaging^58^, and nanographene oxide-HA conjugate for photothermal therapy of melanoma skin cancer^59^. Their results provided the bright hope of using HA on the development of CD44-targeted nanomaterials on diagnostic and therapeutic strategies for cancer.

Hence, we designed the tumor-targeting HA-FeOOH@PPy NRs with a broadband absorption for the PAI-guiding PTT. HA-FeOOH@PPy NRs showed an excellent photothermal effect owing to their strong absorption in the NIR region, and high biocompatible owing to their biocompatible materials. With the tumor targeting ligands, HA-FeOOH@PPy NRs has the well-distribution in the entire tumor with single intratumoural injection, as demonstrated by PAI imaging. The PAI imaging of tumor also showed great promise in the identification of tumor locations, providing a potential tool to guide PTT. According to our results, HA-FeOOH@PPy NRs have shown great binding capacities with CD44-positive breast cancers, and HA-FeOOH@PPy NRs-mediated PTT has led to a selective destruction of CD44-positive breast cancer cells. HA-FeOOH@PPy NRs were investigated *in vivo* to demonstrate their ability to improve the distribution of HA-FeOOH@PPy NRs in tumors. The tumor-bearing mice completely recovered after the combined treatment of HA-FeOOH@PPy NRs and NIR laser irradiation. *In vitro* and *in vivo* test results as well as potential anticancer activity evidenced the potential of HA-FeOOH@PPy NRs in the practical clinic. To the best of our knowledge, this is the first report where HA-FeOOH@PPy NRs were used as novel theragnosis agents for the PAI-guided PTT. Our report highlights the great potential of HA-FeOOH@PPy NRs in the PAI-guided PTT of cancer in future.

## Methods

### Chemicals

Pyrrole (Py, reagent grade, 98%), ferric chloride hexahydrate (FeCl_3_.6H_2_O), sodium phosphate mono basic (NaH_2_PO_4_), polyvinyl alcohol (PVA), dimethyl sulfoxide (DMSO), sodium dodecyl sulfate (SDS), 3-(4,5-dimethylthiazol-2-yl)-2,5-diphenyltetrazolium bromide (MTT), potassium ferrocyanide, and hydrochloric acid were purchased from Sigma-Aldrich (St. Louis, MO, USA), and were used as received during the experiment. Cell staining reagents (trypan blue, PI, and Hoechst 33342) were also purchased from Sigma-Aldrich (St. Louis, MO, USA). Dulbecco’s modified Eagle’s medium (DMEM), fetal bovine serum (FBS), antibiotic, trypsin, and PBS were purchased from HyClone (South Logan, UT, USA). DW was used for all experiments.

### Synthesis of HA-FeOOH@PPy NRs

#### HA-FeOOH@PPy NRs were synthesized by two steps

Step 1- Synthesis of FeOOH nanorod template: The preparation of uniform rod-like shape template FeOOH followed the reported method^60^. Briefly, 100 mL DW containing 0.5406 g FeCl_3_.6H_2_O and 5.4 mg NaH_2_PO_4_ were stirred for 30 min at room temperature and kept in the preheated oven at 100 °C for 24 h. The obtained product was collected by centrifugation (15000 rpm, 15 min), followed by washing three times with DW.

Step 2- Synthesis of HA-FeOOH@PPy NRs: 10 mg SDS was dispersed in 90 mL DW and then 50 mg prepared FeOOH nanorod templates were added to the above solution. 1.50 g PVA dissolved in 20 mL DW at 60 °C for 20 min was also added to the mixed solution. Next, 100 µL pyrrole was added dropwise to the solution together with FeCl_3_.6H_2_O (100 mg in 10 mL) to start polymerization processing. The mixture turned black after a few minutes. After 2 h of polymerization, 20 mL HA (1 mg/mL) was dropped to the above solution. The reaction was kept further for 1 h. Finally, the obtained nanoparticles were collected by centrifugation (15000 rpm, 30 min) and were washed three times with hot water to remove impurities.

### Characterization of HA-FeOOH@PPy NRs

Field-emission transmission electron microscopy (FETEM) images were acquired by a JEOL JEM-2010 microscope (Japan). DLS analysis and zeta potential were examined by an electrophoretic light scattering spectrophotometer (ELS-8000, OTSUKA Electronics Co. Ltd., Japan). The functional groups of the prepared nanoparticles were examined via the Fourier transform infrared spectroscopy (FTIR, Perkin Elmer Inc., USA) over the wavelength range of 400–4000 cm^−1^. UV-Vis spectra were determined by using a UV-Vis spectroscopy (Thermo Biomate 5 Spectrophotometer). The photothermal agents were irradiated by an 808-nm continuous-wave NIR laser (Hi-Tech Optoelectronics Co., Beijing, China). The temperature change was recorded by thermometer (MASTECH, CA, USA) via a thermal fiber. Thermal images were captured using a FLIR i5 infrared camera (Flirt Systems Inc., Portland, USA).

To evaluate the long-term storage stability, the suspension of HA-FeOOH@PPy NRs (60 µg/mL) was stored at 4 °C for 2 months and the UV-Vis absorption spectra of HA-FeOOH@PPy NRs were monitored during this period. To evaluate the dispersion stability, HA-FeOOH@PPy NRs were dispersed in DW, PBS solution, and DMEM containing 10% FBS for 2 months and was monitored the aggregation problems.

### Heating effect evaluation

For measuring the photothermal performance, the HA-FeOOH@PPy NRs were dispersed by DW with different concentrations (10, 20, 30, 50, 70, and 90 µg/mL), and then 1 mL solution was added in a 12-well plate. Each well was directly exposed to NIR laser at the different power densities (0.5, 1, 1.5, and 2 W/cm^2^, 6 min). Temperature changes and thermal images were recorded by the thermometer and infrared camera, respectively.

The photostability of HA-FeOOH@PPy NRs at 100 µg/mL concentration under laser irradiation was evaluated. The suspension was exposed to NIR laser at the power density of 2 W/cm^2^ until reaching the highest temperature and then the solution was naturally cooled to room temperature. While this cycle was repeated three times, the temperature was continuously recorded by the thermometer.

The photothermal conversion efficiency (η) of the HA-FeOOH@PPy NRs was calculated following the previous method^46,48^ (See Supporting Information). The aqueous solution of the HA-FeOOH@PPy NRs (100 µg/mL) was irradiated under the laser (808 nm, 2 W/cm^2^) until a steady state temperature was reached. Next, the laser power was turned off, and the temperature decrease of the aqueous solution was recorded by the thermometer to measure the rate of heat transfer from the HA-FeOOH@PPy NRs solution to the surrounding environment.

### Cell line and cell culture condition

CD44 expressing MDA-MB-231 cell line was bought from Korea Cell Line Bank (Seoul, Korea). DMEM containing 10% (v/v) FBS, 1% (v/v) 100 U/mL penicillin and 100 μg/mL streptomycin are used to culture MDA-MB-231 cells. Cells were grown in a humidified incubator under atmosphere supplemented with 95% air and 5% CO_2_ at 37 °C.

### Cell uptake and *in vitro* cell cytotoxicity assay

The cellular uptake of HA-FeOOH@PPy NRs in the MDA-MB-231 cell was examined by Prussian blue staining (16). The cells (1 × 10^5^ cells/mL) were deposited in 3 mm cell dishes. 1 mL solution of HA-FeOOH@PPy NRs at 100 µg/mL concentration with and without free HA (10 mg/mL) was added to the cell dishes. After 4 h incubation, cold formaldehyde was used to fix the cells for 15 min. Finally, 10% potassium ferrocyanide solution and 20% hydrochloric acid solution were added to the cell plates with the volume ratio 1:1 and incubated for 1 h. The bright field images were taken using an optical microscope.

The MDA-MB-231 cells were seeded at 1 × 10^4^ cells/well in 96-well plates. 100 µL solution of HA-FeOOH@PPy NRs with different concentrations of 0, 20, 30, 50, 70, and 100 µg/mL was added to wells. After 24 hours incubation, 100 µL MTT was added to each well. The cell viabilities were determined by MTT assay.

### *In vitro* photothermal therapy

The MTT assay against MDA-MB-231 cells was used to quantify the effective killing capability of HA-FeOOH@PPy NRs. Briefly, the cells were seeded in a 96-well plate (1 × 10^4^ cells/well). Then, 100 µL solution of HA-FeOOH@PPy NRs at different concentrations were added into wells. After 2 h incubation, the media was discarded and cells were washed three times with PBS to remove unbound nanoparticles. Then, cells were exposed to NIR laser (2 W/cm^2^, 6 min). After 2 h of further incubation, cell viability was determined by the MTT assay.

For qualitative evaluation, the killed cells were also co-stained by Hoechst 33342 and PI. Briefly, the cells were seeded in a 12-well plate (1 × 10^5^ cells/well). After 24 h incubation, the cells were divided into four groups with different treatment methods: PBS (group I); PBS plus NIR laser (group II); HA-FeOOH@PPy NRs (group III) and HA-FeOOH@PPy NRs plus NIR laser (group IV). After 2 h incubation, the cells in group II and IV were exposed to NIR laser at the power density of 2 W/cm^2^ for 6 min. Then, all cells were co-stained with Hoechst 33342 and PI after further 2 h incubation. Finally, the fluorescent images of all groups were taken by a fluorescence microscope (Leica Microsystems GmbH, Wetzlar, Germany).

### Animal and tumor model

Female BALB/c nude mice (6 weeks, 18–20 g) were bought from Orient Bio Inc. (Gyeonggi-Do, Korea). All described methods were approved by Pukyong National University Animal Care and Use Committee (Korea), and performed in accordance with relevant guidelines and regulations for the care and use of laboratory animals. The mice were kept in standard stainless steel cages (3 mice/cage). 100 µL cell suspension of 5 × 10^6^ MDA-MB-231 cells was subcutaneously injected into the right leg of the mice. The digital caliper was used to measure the width and length of the tumor. When the tumor volume reached 125 to 135 mm^2^, the mice could be used for *in vivo* experiments.

### *In vitro* and *in vivo* photoacoustic therapy

We have recently build the PAM system to monitor the presence of nanoparticles in mouse tumor model^61^. The PAM system used a single-mode optical fiber with polarization-maintaining (PM-S405-XP) to deliver light from a diode pumped passively Q-switched solid state laser of 532 nm (SPOT-10-100-532). The light in the output of the single-mode fiber was used to excite a sample to generate PA waves with an incident laser fluence below 13.3 mJ/cm^2^. To maximize the sensitivity of detection, the light was aligned confocally with a spherically focused transducer through a custom-made acoustic and optical beam combiner (AOBC).

A two-axis linear stage provides the sample’s volume imaging via a raster scan of the AOBC along xy-plane. A focused transducer of 10 MHz (Olympus) was utilized to capture the generated PA signals. Then, a data acquisition system synchronizing with the laser system was used to amplify, digitize, and store the captured PA signals. Lastly, the Hilbert transformation was applied to convert the stored PA signals to PA images. In the scanning plane, the scan’s step sizes were set as 2 and 20 μm for the x and y axes, respectively. For a 12 × 12-nm field of view, the acquisition time was about 25 min. Photoacoustic information of samples was acquired under the full spectrum of SRS source.

*In vitro* photoacoustic imaging: the human tissue-mimicking PVA phantom was fabricated following a previous report^62^. The MDA-MB-231 cells were treated with different concentrations (50, 100, and 200 μg/mL) of HA-FeOOH@PPy NRs. The control cells and HA-FeOOH@PPy NRs-treated cells were mixed with 4% gelatin and loaded into the wells (50 μL each/well) of the phantom.

*In vivo* photoacoustic imaging: two nude female mice with the same tumor volume were used to obtain the PA tumor images. The mice were intratumourally injected with HA-FeOOH@PPy NRs (70 μL of 1 mg/mL). FeOOH@PPy NRs with the same concentration which is non-targeting tumor nanoparticles were used as a control. 5 h after injection, the PA images of the tumors were acquired by the PAM system.

### *In vivo* photothermal therapy

When the tumor volume reached 125 to 135 mm^2^, the mice were divided into four groups (n = 3) for treatment with PBS (group I); PBS plus NIR laser (group II); HA-FeOOH@PPy NRs (group III); and HA-FeOOH@PPy NRs plus NIR laser (group IV).The mice were intratumourally injected with 70 μL of 1 mg/mL HA-FeOOH@PPy NRs. Following that, the mice in groups II and IV were treated with NIR laser irradiation at 2 W/cm^2^ for 6 min. The temperature changes occurring in the irradiated tumor region was continuously measured with a thermal imaging camera. Mice body weight and tumor volume were measured on the following days. The tumor volume was determined by formula V = ½ (L·W^2^), where L is length and W is the width of the tumor.

### Histological analysis

After 17 days, the mice from each group were sacrificed and five major organs including the heart, liver, spleen, lung, and kidney were collected. After rinsed three times with physiological saline, each organ was immediately immersed in 10% formalin for fixation. After immersing for 24 h, the tissues were implanted in paraffin, sectioned into 4 μm thick sections, then stained with hematoxylin and eosin and the slices were visualized by the light microscope.

## Electronic supplementary material

Supplementary Information
