# Supplementary material for: Photoacoustic Imaging-Guided Photothermal Therapy with Tumor-Targeting HA-FeOOH@PPy Nanorods
Source: Sci Rep. 2018 Jun 11;8:8809. doi: 10.1038/s41598-018-27204-8 (PMC5995888; doi:10.1038/s41598-018-27204-8)
Supplement: Supplementary file 1 — Supplementary Information [file 41598_2018_27204_MOESM1_ESM.docx]

Photoacoustic Imaging-Guided Photothermal Therapy with Tumor-Targeting HA-FeOOH@PPy Nanorods

*Thi Tuong Vy Phan^1,2^, Nhat Quang Bui^1^, Soon-Woo Cho^4^, Subramaniyan Bharathiraja^2^, Madhappan Santha Moorthy^2^, Panchanathan Manivasagan^2^,* [*Sudip Mondal*](http://www.mdpi.com/search?authors=Sudip%20Mondal&orcid=)*^2^, Chang-Seok Kim^2^ and Junghwan Oh,^12,3,*^*

^1^Interdisciplinary Program of Biomedical Mechanical & Electrical Engineering, Pukyong National University, Busan, 48513, Republic of Korea

^2^Center for Marine-Integrated Biomedical Technology, Pukyong National University, Busan, 48513, Republic of Korea

^3^Department of Biomedical Engineering, Pukyong National University, Busan, 48513, Korea Republic of Korea

^4^Department of Cogno-Mechatronics Engineering, Pusan National University, Busan, 46241, Republic of Korea

* Corresponding author:

Prof. Junghwan Oh^1,2,3*^

Email: [jungoh@pknu.ac.kr](mailto:jungoh@pknu.ac.kr) (J. Oh). Tel: +82-51-629-5771; Fax: +82-51-629-5779.

Supplementary experimental section

| Nanoparticles | Hydrodynamic  Diameter (nm) | Zeta Potential (mV) |
| --- | --- | --- |
| FeOOH | 45 | +3.16 |
| HA-FeOOH@PPy NRs | 82 | -11.63 |

### Table S1: Size and zeta potential of the synthesized nanoparticles


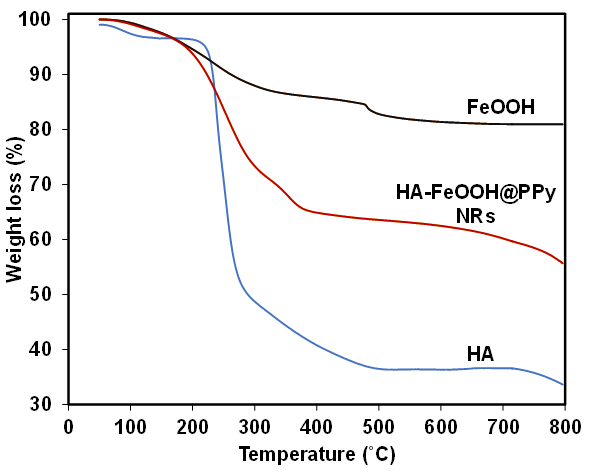


Figure S1: TGA analysis of FeOOH, HA-FeOOH@PPy NRs, and HA.

Figure S2: Microscopy images with Prussian blue staining showing co-treatment of free HA (10 mg/mL) reduces intracellular uptake of HA-FeOOH@PPy NRs into MDA-MB-231 cells. Incubation time is 2 h.

Figure S3: (A) The real-time temperature record of 3 heating/cooling cycles of 100 µg/ml HA-FeOOH@PPy NRs under on/off laser experiment. (B) The UV-Vis absorption of HA-FeOOH@PPy NRs before and after irradiation.

Figure S4: (A) The UV-Vis-NIR absorption spectra of HA-FeOOH@PPy NRs during long-term storage test. (B) The photos of HA-FeOOH@PPy NRs in different media after 2 months storage.


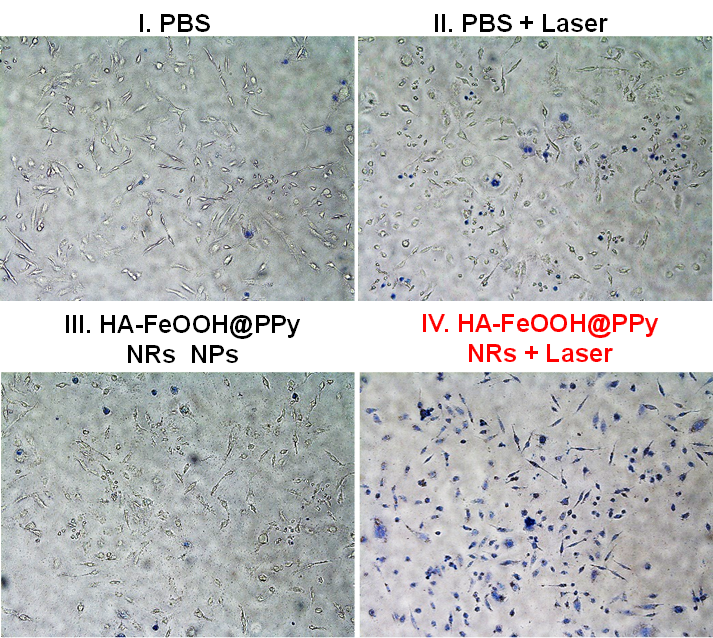


Figure S5: Trypan blue staining of MDA-MB-231 cells treated with 100 μg/mL HA-FeOOH@PPy NRs plus NIR light irradiation (2 W/cm^2^, 6 min).


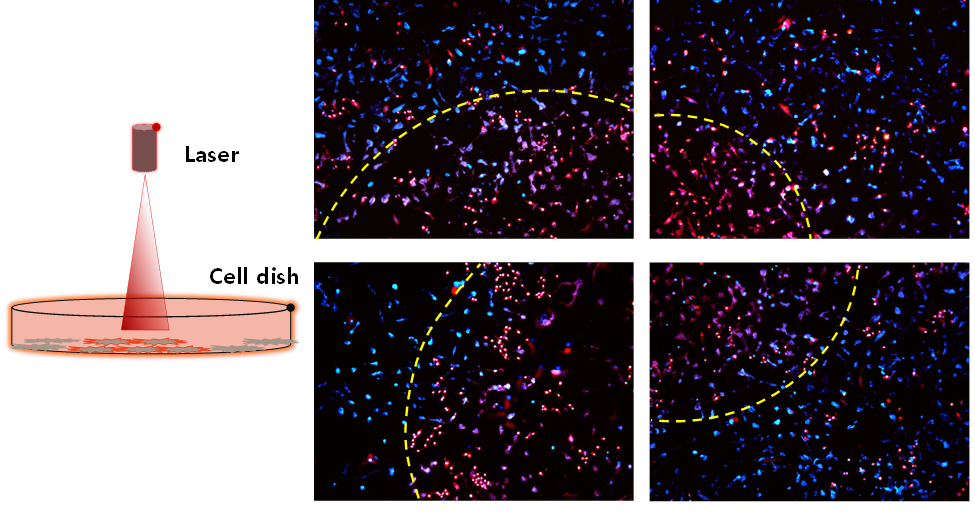


Figure S6: Laser spot of MDA-MB-231 cells treated with 100 μg/mL HA-FeOOH@PPy NRs plus NIR light irradiation (2 W/cm^2^, 6 min).

Figure S7. (A) Photograph of a tissue mimicking PVA phantom showing a top view structure. (B) Photoacoustic images of MDA-MB-231 cells which untreated and treated with diﬀerent concentrations of HA-FeOOH@PPy NRs (200, 100, and 50 μg/mL).


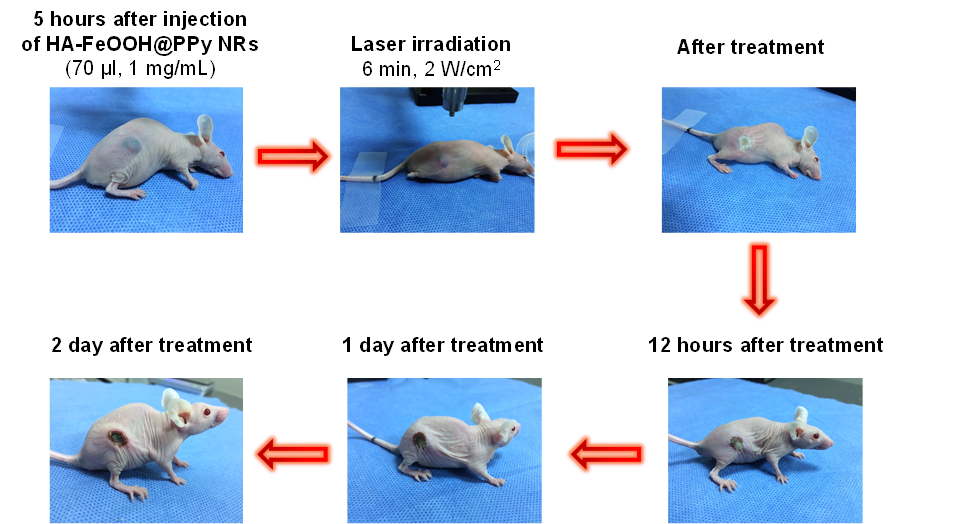


Figure S8: Treatment processing


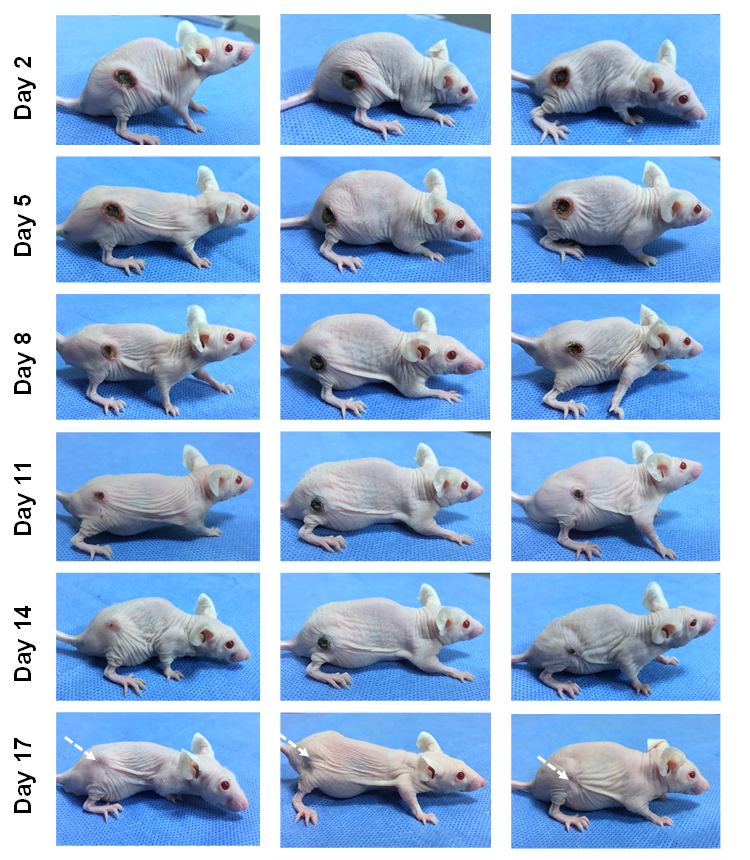


Figure S9: Photographs documenting tumor development at different days in live mice treated under treatment conditions on group IV (HA-FeOOH@PPy NRs + Laser).


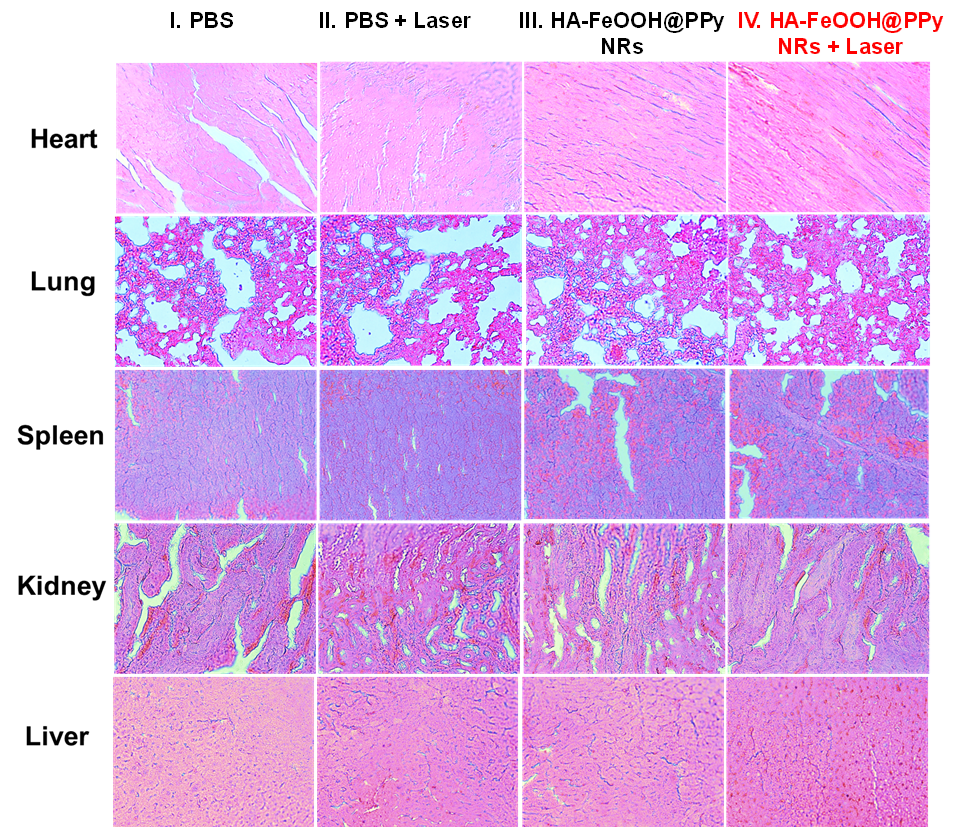


Figure S10: H&E-stained histological images of five organ tissues (heart, liver, spleen, lung and kidney) from all groups after PTT for 17 days.

Calculation of the photothermal conversion efficiency

According to the previous method[^1^](#_ENREF_1)^,^[^2^](#_ENREF_2), the photothermal conversion efficiency (*η)* of the HA-FeOOH@PPy NRs was computed using Eq. (1):

*η* = $\frac{\boldsymbol{hS (}{\boldsymbol{T}_{\boldsymbol{Max}}\boldsymbol{- T}}_{\boldsymbol{Surr}}\boldsymbol{)}\boldsymbol{-Q}_{\boldsymbol{Dis}}}{\boldsymbol{I(1-}\boldsymbol{10}^{\boldsymbol{-}\boldsymbol{A}_{\boldsymbol{808}}}\boldsymbol{)}}$ (1)

in which *h* is the heat transfer coefficient and *S* is the surface area of the container. The maximum steady temperature (*T_Max_)* of the solution of the HA-FeOOH@PPy NRs was 67.7 ^o^C and ambient temperature (*T_Surr_*) was 22 ^o^C. So, the temperature change (*T_Max_*-*T_Surr_*) of the HA-FeOOH@PPy NRs solution was 47.7 ^o^C. The laser power density was 2 W/cm^2^, the diameter of container was 1 cm. So, the laser power (*I*) was 1.57 W. The absorbance of the HA-FeOOH@PPy NRs at 808 nm (*A*_808_) is 0.37. *Q_Dis_* expresses heat dissipated from the light absorbed by the solvent and container.

The value of *hS* can be obtained from Eq. (2), as follows:

*hS* = $\frac{m_{D}C_{D}}{{}_{S}}$ (2)

where *m* is 1 g and *C* is 4.186 J/g·℃. The sample system time constant *τ_s_* can be computed using Eqs. (3)-(4) as:

*θ =*$\frac{{T-T}_{Max}}{T_{Max}{-T}_{Sur}}$ (3)

t=-*τ_s_ ln* (*θ* ) (4)

in which *θ*  is a dimensionless parameter.

Time constant for heat transfer *τ_s_* was calculated as 519.44 s by applying the linear time data from cooling period (Figure S11B). Thus, according to Eq. (4), *hS* was computed as 8.089 mW/ ^o^C. *Q_Dis_* was measured independently to be 34.88 mW.

Finally, by substituting all obtained parameters into Eq. (1), the 808 nm laser heat conversion efficiency (*η*) of the HA-FeOOH@PPy NRs was calculated to be 37.17%, which is significantly higher than that of widely used gold nanorods (21%)[^1^](#_ENREF_1)^,^[^3^](#_ENREF_3).


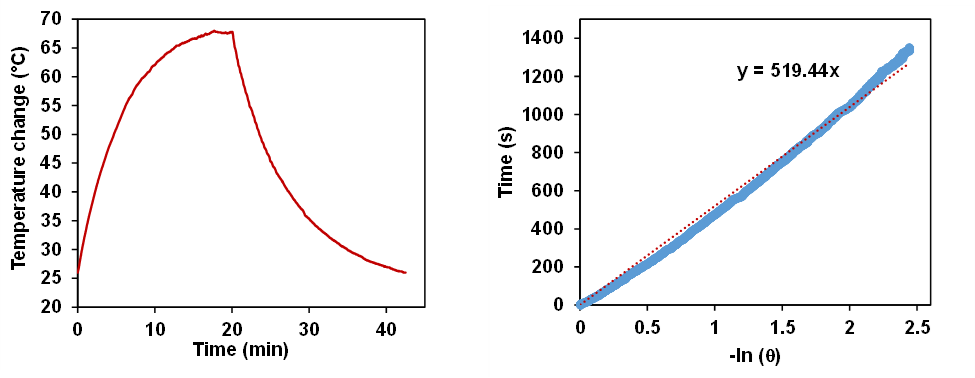


Figure S11: (A) Photothermal heating curve of the aqueous solution of HA-FeOOH@PPy NRs (100 µg/mL) upon 808 nm laser irradiation (2 W/cm^2^), and then the laser was shut off. (B) Linear time data versus –ln (θ) obtained from the cooling period from Figure S11A.

1 Liu, X. *et al.* Facile synthesis of biocompatible cysteine-coated CuS nanoparticles with high photothermal conversion efficiency for cancer therapy. *Dalton transactions (Cambridge, England : 2003)* 43, 11709-11715, doi:10.1039/c4dt00424h (2014).

2 Tian, Q. *et al.* Hydrophilic Cu9S5 Nanocrystals: A Photothermal Agent with a 25.7% Heat Conversion Efficiency for Photothermal Ablation of Cancer Cells in Vivo. *ACS Nano* 5, 9761-9771, doi:10.1021/nn203293t (2011).

3 Wang, B. *et al.* Rose-bengal-conjugated gold nanorods for in vivo photodynamic and photothermal oral cancer therapies. *Biomaterials* 35, 1954-1966, doi:https://doi.org/10.1016/j.biomaterials.2013.11.066 (2014).
